# Herbal Components of a Novel Formula PSORI-CM02 Interdependently Suppress Allograft Rejection and Induce CD8+CD122+PD-1+ Regulatory T Cells

**DOI:** 10.3389/fphar.2018.00088

**Published:** 2018-02-12

**Authors:** Chuanjian Lu, Huazhen Liu, Xiaowei Jin, Yuchao Chen, Chun-Ling Liang, Feifei Qiu, Zhenhua Dai

**Affiliations:** ^1^Section of Immunology and Joint Immunology Program, Guangdong Provincial Hospital of Chinese Medicine, The Second Affiliated Hospital of Guangzhou University of Chinese Medicine, Guangzhou, China; ^2^Department of Integrative Chinese-Western Medicine, The Third Affiliated Hospital of Kunming Medical University, Kunming, China

**Keywords:** herbal medicine, immunosuppression, immunoregulation, transplantation, Treg

## Abstract

A recipient usually rejects a transplanted organ and thus needs immunosuppressive treatments to prevent rejection. Achieving long-term allograft survival without continuous global immunosuppression is highly desirable in transplantation as long-term immunosuppression causes various side effects. Therefore, it is necessary to search for medicine with potentially less side effects. Traditional Chinese medicine PSORI-CM01 (Yin Xie Ling), a formula with seven natural herbs, has been used to treat patients with psoriasis. Here, we investigated a “sharpened” formula, PSORI-CM02 consisting of only five herbs from PSORI-CM01: Curcumae rhizoma, Radix paeoniae rubra, Rhizoma smilacis glabrae, Mume fructus, and Sarcandrae herba. We examined whether or not PSORI-CM02 would suppress alloimmunity and found that PSORI-CM02 significantly inhibited murine skin allograft rejection and reduced graft-infiltration of CD3+ T cells. Interestingly, omitting any single herbal component rendered the whole formula ineffective in suppression, indicating that these herbal components exert their effects cooperatively as a whole. Moreover, PSORI-CM02 increased CD8+CD122+PD-1+ Treg frequency with CD4+FoxP3+ Tregs remaining unchanged in recipient mice, whereas CsA reduced CD4+FoxP3+ Treg frequency. PSORI-CM02 also hindered CD11c+ DC maturation posttransplantation. Importantly, PSORI-CM02-induced CD8+CD122+PD-1+ Tregs were more potent in suppression of allograft rejection in Rag-/- mice than control Tregs. On the other hand, PSORI-CM02 suppressed T cell proliferation *in vitro* and reduced their phosphorylation of P70S6K and P50/P65, suggesting that it inhibits both mTOR and NFκB signaling pathways. It also increased IL-10 production while reducing IFNγ level in the supernatant of activated T cells co-cultured with CD8+CD122+PD-1+ Tregs. Furthermore, HPLC fingerprinting ruled out that PSORI-CM02 contained CsA or rapamycin. PSORI-CM02 also did not cause any illness and toxic injury in recipient mice. Thus, we demonstrate that PSORI-CM02 formula suppresses allograft rejection without toxicity.

## Introduction

Achieving long-term allograft survival without continuous use of immunosuppressive agents is a long-term goal in transplantation, because continuous immunosuppression causes a variety of adverse reactions, including infections, and tumors. Furthermore, some immunosuppressive agents can inhibit the development and function of regulatory T cells (Tregs) and hinder tolerance induction. In particular, CsA blocks IL-2 expression (Zeiser et al., 2006; Kang et al., 2007; Noris et al., 2007) and consequently compromises survival and suppressive capacity of Tregs (Furtado et al., 2002; Malek et al., 2002; Thornton et al., 2004). Hence, prolongation of allograft survival via short-term treatments with non-toxic and effective medicine is meaningful.

Chinese medicine PSORI-CM01 (Yin Xie Ling), a formula with seven herbs, has been well known in China and widely used to treat autoimmune diseases by local doctors for a long period of time. In fact, use of PSORI-CM01 to treat autoimmune diseases in clinic has been largely undocumented in China. In documented clinical practice, PSORI-CM01 has been effective in treating autoimmune psoriasis with no any significant side effect (Lu et al., 2012; Parker et al., 2014). In a randomized, double-blinded and multicentral clinical trial, it was shown that Yin Xie Ling significantly improved PASI scores and relapse rates in psoriasis vulgaris (Deng et al., 2017). It also reduced PASI scores and decreased serum TNFα level in patients with psoriasis vulgaris (Dai Y.J. et al., 2014). Our previous animal studies have demonstrated that PSORI-CM01 exerts anti-inflammatory effects via inhibiting production of proinflammatory cytokines and chemokines (Wei et al., 2016; Yao et al., 2016; Han et al., 2017). However, it’s unknown whether PSORI-CM01 can inhibit allograft rejection. In an effort to minimize herbs in the formula that could be unnecessarily taken by patients, we omitted two herbs, Liquorice and Lithospermum, in PSORI-CM01 formula with the rest of five herbs remaining, and renamed it as PSORI-CM02.

We determined whether the “sharpened” PSORI-CM02 formula would suppress allograft rejection. We found that PSORI-CM02 significantly delayed murine skin allograft rejection. Moreover, it suppressed proliferation of T cells and inhibited both mTOR and NFκB signaling pathways. Furthermore, PSORI-CM02 increased CD8+CD122+PD-1+ Treg frequency and enhanced their suppressive capacity as well. HPLC analyses demonstrated that PSORI-CM02 formula did not contain CsA and rapamycin while treatments with PSORI-CM02 also did not cause any toxic reaction in recipient mice. Omission of any single herbal component rendered the whole formula ineffective in suppression of allograft rejection. Therefore, we proved the necessity to simultaneously utilize several herbs in order for a herbal formula to take effect through mechanistic studies on immunology instead of results-oriented theory of TCM.

## Materials and Methods

### Mice and Antibodies

Wild-type BALB/c and C57BL/6 mice were purchased from Guangdong Medical Laboratory Animal Center (Fushan, Guangdong, China). Rag1-/- (B6) and Thy1.1 (B6.PL-Thy1^a^) mice were purchased from the Jackson Laboratory (Bar Harbor, ME, United States). All mice were housed in a specific pathogen-free (SPF) environment. The animal protocol was approved by the Institutional Animal Care and Use Committee of Guangdong Provincial Academy of Chinese Medical Sciences. CsA (Novartis, Basel, Switzerland) was obtained from Department of Pharmacy, Guangdong Provincial Hospital of Chinese Medicine (Guangzhou, China). Anti-CD4-PE, anti-CD3-FITC, anti-CD8-PE, anti-CD8-FITC, anti-Thy1.1-PerCP, anti-CD122-PE, and anti-PD-1-APC Abs were purchased from BD Biosciences (San Jose, CA, United States). Anti-Foxp3-APC Ab, its related intracellular fixation/permeabilization kits, and purified anti-CD3 plus anti-CD28 mAb were purchased from eBioscience (San Diego, CA, United States). Purified antibodies against p70S6K, Phospho-p70S6K, Phospho-Rel B, and Phospho-P50/52/65 were bought from Cell Signaling Technology (Boston, MA, United States).

### Treatments of Mice

PSORI-CM02 was dissolved in distilled water and orally administered at 2–6 g/kg daily for 2–4 weeks post-transplantation or until an allograft was rejected. Meanwhile, CsA was also administered i.p. at 20 mg/kg/day for 2–4 weeks. At the end of experiments, all samples were collected. The dosage of PSORI-CM02 (6 g/kg/day) was calculated based on the clinical usage that did not cause side effects in patients (Lu et al., 2012; Parker et al., 2014).

### Skin Transplantation

Skin donors were 7- to 8-week-old wild-type BALB/c male mice, and skin graft recipients were 7- to 8-week-old C57BL/6 male mice. Full-thickness trunk skin was transplanted to the dorsal flank area of recipient mice and secured with the bondage of Band-Aid (Johnson Johnson, New Brunswick, NJ, United States). Skin graft rejection was defined as graft necrosis greater than 90%, as described in our previous publication (Dai et al., 2004).

### Preparation of PSORI-CM02

PSORI-CM02 formula and individual herbs of clinical grade were obtained from Guangdong Provincial Hospital of Chinese Medicine and produced by Guangdong Kangmei Pharmaceutical Company Ltd. (Guangdong, China). Its formula includes five herbs: Curcumae rhizoma, Radix paeoniae rubra, Rhizoma smilacis glabrae, Mume fructus, and Sarcandrae herba with a weight ratio of 2:3:5:3:5. All herbal decoctions were prepared according to standard procedures (Chen et al., 2013), and all of the procedures were in accordance with the rule and regulation in 2010 Edition of China Pharmacopoeia. Water extracts were then concentrated and dried out with a rotary evaporator under vacuum.

### Cell Surface and Intracellular Staining for FACS Analysis and Cell Sorting

B6 mice were transplanted with a BALB/c skin graft and treated with PSORI-CM02 as described above. Draining lymph node and spleen cells from recipient mice were pooled after lysing red blood cells. Cells were stained for surface markers with anti-CD4-PE, anti-CD8-PE, anti-CD8-FITC, anti-CD122-FITC, or anti-PD-1-APC, and then intracellular markers in some groups with anti-FoxP3-APC using intracellular fixation/permeabilization kits. CD4+Foxp3+ and CD8+CD122+PD-1+ Tregs finally were enumerated by FACS anassssslyses.

To purify CD3+CD122- T cells and CD8+CD122+PD-1+ Tregs for adoptive transfer experiments, LN and spleen cells were stained with anti-CD3-FITC/anti-CD122-PE or anti-CD8-FITC/anti-CD122-PE/anti-PD-1-APC Abs. CD3+, CD3+CD122- T cells, CD8-CD122+ cells or CD8+CD122+PD-1+ Tregs were then sorted via FACSAria III (BD Biosciences). The purity of the sorted cells was typically >95%.

### Hematoxylin–Eosin (HE) and Immunohistochemical (IHC) Staining

Skin grafts were fixed in 4% neutral formaldehyde for 24 h and processed for paraffin embedding. Some sections (3.5 mm) were stained with HE while others were incubated with primary monoclonal anti-CD3 antibodies at 4°C overnight. After incubated with HRP-anti-mouse IgG and colored with 3′-diaminobenzidene (DAB, Sigma-Aldrich), sections were counterstained by hematoxylin.

### Proliferation Assay and Measurement of Cytokines in the Supernatant

FACS-sorted CD3+ T cells were labeled with 2 μM CFSE (Invitrogen, Karlsruhe, Germany) for 10 min at room temperature. Subsequently, cells (2 × 10^5^ cells/well) were cultured in 96-well plates in complete RPMI-1640 medium (supplemented with 10% fetal bovine serum, 100 IU/mL penicillin and 100 mg/mL streptomycin) and stimulated with plate-bound anti-CD3 (5 μg/ml) plus soluble anti-CD28 mAb (2.5 μg/ml). Cells were also treated with PSORI-CM02 (1 mg/ml) or CsA (0.1 mg/ml) and cultured at 37°C with 5% CO_2_ for 96 h. In a separate MLR assay, purified C57BL6-derived T cells were labeled with CFSE and then cultured with irradiated donor splenocytes (stimulators) from Balb/C mice at a ratio of 1:1 for 96 h. Finally, cell proliferation was analyzed through a FACSCalibur (BD Biosciences) while IL-10 and IFNr in the supernatant were measured via ELISA according to the manufacturer’s instructions (Boster, Wuhan, China).

### Cell Apoptosis Analysis

Apoptosis was measured using an Annexin V-FITC kit (Invitrogen, Carlsbad, CA, United States) according to the manufacturer’s instructions. Briefly, cells were collected after the culture with PSORI-CM02 or CsA for 96 h. Cells were then stained with Annexin V-FITC and propidium iodide (PI) solutions. Apoptotic cells finally were determined by FACS analyses.

### High Performance Liquid Chromatography (HPLC) Analysis

All standard samples, including chlorogenic acid, astilbin, isofraxidine, paeoniflorin (Solarbio, China), CsA and rapamycin (Sigma-Aldrich, United States), were dissolved with methanol while PSORI-CM02 was dissolved with deionized water. The samples were put into HPLC system (Agilent 1200 HPLC system, Santa Clara, CA, United States), followed by separation on the chromatographic column C18 (4.6 mm × 250 mm, 5 μM, ACE, Scotland). 10 μL sample solution was injected into HPLC using C18 column in each running for the analysis. The mobile phase consisted of deionized water with 0.1% formic acid (phase A) and acetonitrile with 0.1% formic acid (phase B). The gradient elution program was described as follows: 10–1% B at 0–5 min, 10–20% B at 5–10 min, 20–40% B at 10–15 min, 40–95% B at 15–50 min, 95–100% B at 50–55 min. The flow rate was 1.0 mL/min, and the detection wavelength was set at 334 nm.

### Western Blotting

Cell protein was extracted using RIPA buffer (50 mM Tris pH 7.5, 150 mM NaCl, 1% Triton X-100, and 5 mM ethylenediaminetetraacetic acid). Protein concentration was measured using the BCA Kit (Pierce, IL, United States). The protein samples (40 ug/each) were separated by 8% sodium dodecyl sulfate (SDS)-polyacrylamide gel electrophoresis, and then transferred to a nitrocellulose membrane. The blots were probed using primary antibodies against murine p70S6K, Phospho-p70S6K, Phospho-Rel B and Phospho-P50/52/65. Blots were then incubated with horseradish peroxidase-conjugated goat anti-rabbit Ab at a dilution of 1:10000. Blots were finally detected using a Bio-Rad Gel imaging system.

### Statistical Analysis

Comparisons of the mean were performed using ANOVA. The analysis of graft survival was conducted using a Kaplan–Meier method (log-rank test). All analyses were performed using Prism-6 software (GraphPad Software, La Jolla, CA, United States). Data were presented as mean ± SD. A value of *P* < 0.05 was considered statistically significant.

## Results

### Treatments with PSORI-CM02 Prolong Skin Allograft Survival and Reduce CD3+ T Cell Infiltration in an Allograft

**FIGURE 1:**
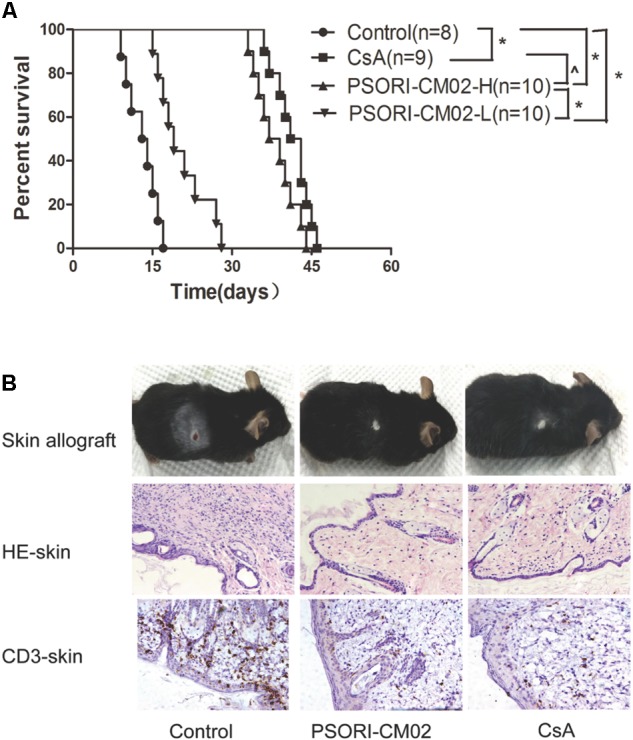
PSORI-CM02 prolongs skin allograft survival and reduces CD3+ T cell infiltration in an allograft. Skin grafts derived from donor Balb/C mice were transplanted to C57BL/6 mice, which were then treated with PSORI-CM02 (PSORI-CM02-H = 6 g/kg/day and PSORI-CM02-L = 2 g/kg/day) or CsA (20 mg/kg/day) for 4 weeks or until rejection. **(A)** Skin allograft rejection was observed with 8–10 transplants per group. **(B)** Also shown was a representative of rejected or accepted skin allografts, cellular infiltration (HE) and infiltration of CD3+ T cells (IHC staining) for each group 2 weeks post-treatment and post-transplantation. One representative from 4 to 5 mice per group is shown (^∗^ represents *P* < 0.05 while ˆ indicates *P* > 0.05). The experiments were repeated twice with the similar results.

Given that PSORI-CM01 formula has been shown to effectively treat autoimmune psoriasis, we asked whether the “sharpened” formula PSORI-CM02 would suppress allograft rejection. C57BL/6 mice were transplanted with a skin graft from Balb/C mice and treated with PSORI-CM02 or CsA. As shown in **Figure 1A**, we found that PSORI-CM02, at either low or high doses, significantly prolonged skin allograft survival compared to the control group (median survival time, MST = 19 [low dose] vs. 13 days and 39 [high dose] vs. 13 days, *n* = 8–10, both *P* < 0.05) while high doses of PSORI-CM02 further extended skin allograft survival compared to the low doses (MST = 39 vs. 19 days, *P* < 0.05). Moreover, PSORI-CM02, when administered at high doses, was as effective in prolongation of allograft survival as CsA (20 mg/kg/day) (MST = 39 vs. 41 days, *P* > 0.05). Interestingly, this new formula did not significantly extend skin allograft survival when any single herbal component was omitted (**Table 1**), suggesting that PSORI-CM02 formula works through cooperation between all herbs. PSORI-CM02 prolonged skin allograft survival just as effectively as PSORI-CM01 (MST = 39 vs. 40 days, *P* > 0.05) (**Table 1**). We chose the high doses of 6 g/kg/day for PSORI-CM02 according to the clinical usage of PSORI-CM01 that did not result in any major side effect in patients (Lu et al., 2012; Parker et al., 2014) while our studies demonstrated that this dosage did not cause any illness and toxic injury to a murine kidney or liver (HE staining, Supplementary Figure S1). Biochemical laboratory tests of renal and liver function were also normal (Supplementary Table S1). Also shown in **Figure 1B** were representatives of a rejected skin graft from a control recipient and an accepted skin graft from a PSORI-CM02- or CsA-treated recipient 2 weeks post-transplantation. HE staining and IHC revealed that either PSORI-CM02 or CsA obviously attenuated both generally cellular and CD3+ T cell infiltration in a skin allograft compared to the control group (**Figure 1B**), suggesting that PSORI-CM02 is as efficient as CsA in suppression of cellular alloimmunity.

**Table 1:** PSORI-CM02, but not the decomposed formula, prolongs skin graft survival.

Formula	*N*	Survival days	MST (days)
Control	7	9, 10, 11, 13, 14, 15, 16	13
Full PSORI-CM01: L, Lm, CR, RPR, RSG, MF, SR	8	33, 34, 38, 39, 41, 43, 46, 47	40^∗^
Full PSORI-CM02: CR, RPR, RSG, MF, SR	8	34, 35, 36, 38, 39, 40, 41, 43	39^∗#^
Decomposed formula 1: CR, RPR, RSG, MF	7	9, 11, 12, 14, 15, 16, 17	14
Decomposed formula 2: CR, RPR, RSG, SR	7	11, 13, 14, 15, 16, 17, 19	15
Decomposed formula 3: CR, RPR, MF, SR	8	9, 10, 12, 14, 16, 17, 18, 20	15
Decomposed formula 4: CR, RSG, MF, SR	7	11, 13, 14, 16, 17, 18, 20	16
Decomposed formula 5: RPR, RSG, MF, SR	9	9, 10, 11, 13, 14, 15, 17, 19, 20	14

*Skin allograft rejection was observed with 7–9 transplants per group after treatments with full or each decomposed formula at doses of 6 g/kg/day. While the full formula contained five herbs, each decomposed formula missed one herb, ending up with a total of five decomposed formulas of four herbs per formula. Abbreviations: Liquorice (L), Lithospermum (Lm), Curcumae Rhizoma (CR), Radix Paeoniae Rubra (RPR), Rhizoma Smilacis Glabrae (RSG), Mume Fructus (MF), and Sarcandrae herba (SR). ^∗^*P* < 0.05 compared to control and ^#^*P* > 0.05 compared to PSORI-CM01.*

### PSORI-CM02 Does Not Contain an Immunosuppressant Cyclosporine or Rapamycin

Since PSORI-CM02 inhibited alloimmune responses and allograft rejection, it is imperative to rule out that they contain an ingredient of a conventional immunosuppressant, such as CsA or rapamycin. To this end, we generated HPLC fingerprints of PSORI-CM02 formula with control samples, including both CsA and rapamycin. As shown in Supplementary Figure S2, the histogram peaks for PSORI-CM02 were overwhelmingly located within 24 min while those for CsA and rapamycin fell in the range of 40–50 min, suggesting that PSORI-CM02 does not contain conventional immunosuppressive agents CsA and rapamycin.

### PSORI-CM02 Inhibits CD11c+ DC Maturation after Transplantation

**FIGURE 2:**
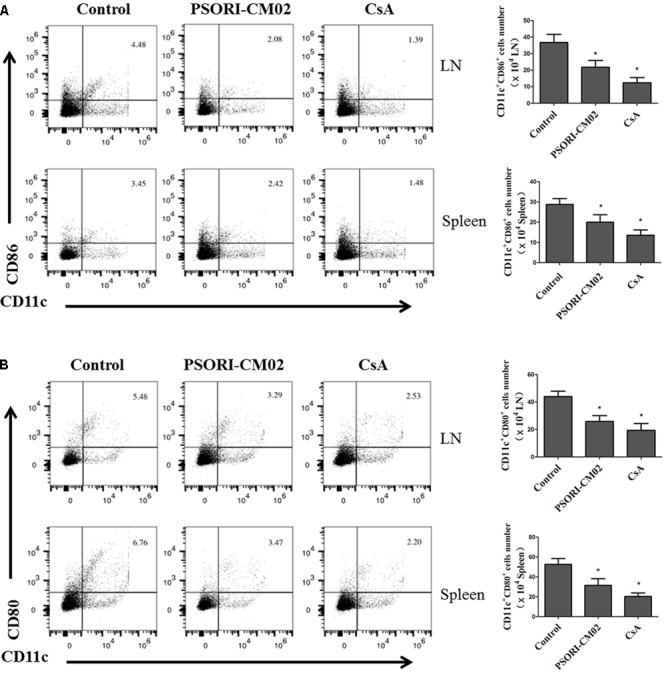
PSORI-CM02 hinders DC maturation posttransplantation. Draining LN and spleen cells were isolated and CD80+CD11c+ or CD86+CD11c+ DCs were quantified by FACS analysis 2 weeks after transplantation and treatments with high doses of PSORI-CM02. The percentages of CD86+CD11c+ **(A)** and CD80+CD11c+ **(B)** DCs in LN and spleen cells of the recipient mice were analyzed. Data are presented as mean ± SD with 4–5 mice per group. One representative experiment is shown. The experiments were repeated twice with similar results. (^∗^ represents comparisons with control, *P* < 0.05).

Since, we found that PSORI-CM02 suppressed allograft rejection, we first asked whether it would inhibit alloimmune responses by hindering DC maturation. Draining LN and spleen cells were isolated and both CD80+CD11c+ and CD86+CD11c+ DCs were enumerated by FACS analyses 2 weeks following skin transplantation and treatments with PSORI-CM02 or CsA. As shown in **Figure 2**, either PSORI-CM02 or CsA significantly reduced the frequency of CD80+CD11c+ DCs (mean ± SD = 31.49 ± 6.66 vs. 52.67 ± 5.76 and 20.31 ± 3.56 vs. 52.67 ± 5.76, both *P* < 0.05, × 10^4^ in spleens; 25.83 ± 4.20 vs. 43.96 ± 3.94 and 19.37 ± 4.99 vs. 43.96 ± 3.94, both *P* < 0.05, × 10^3^ in LNs) and CD86+CD11c+ DCs (mean ± SD = 19.79 ± 3.70 vs. 28.77 ± 2.86 and 13.63 ± 2.54 vs. 28.77 ± 2.86, both *P* < 0.05, × 10^4^ in spleens; 21.77 ± 4.16 vs. 36.67 ± 5.04 and 12.41 ± 3.08 vs. 36.67 ± 5.04, both *P* < 0.05, × 10^3^ in LNs) in both draining LNs and spleens of recipient mice, suggesting that PSORI-CM02 formula suppresses DC maturation.

### PSORI-CM02 Increases CD8+CD122+PD-1+, But Not CD4+FoxP3+, Treg Frequency

**FIGURE 3:**
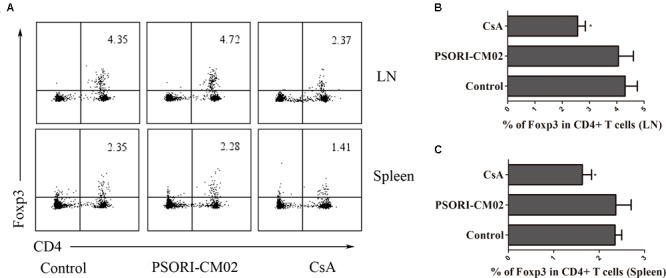
PSORI-CM02 does not promote CD4+FoxP3+ Treg generation. Draining LN and spleen cells were isolated and CD4+FoxP3+ Tregs were measured by FACS 2 weeks after transplantation and treatment with high doses of PSORI-CM02. One representative of dot plots per group was shown **(A)**. The percentages of CD4+FoxP3+ Tregs in LN **(B)** or spleen cells **(C)** were analyzed. Data are presented as mean ± SD from 4 to 6 mice per group. Shown is a representative of three separate experiments with similar results. (^∗^ represents comparisons with control, *P* < 0.05).

**FIGURE 4:**
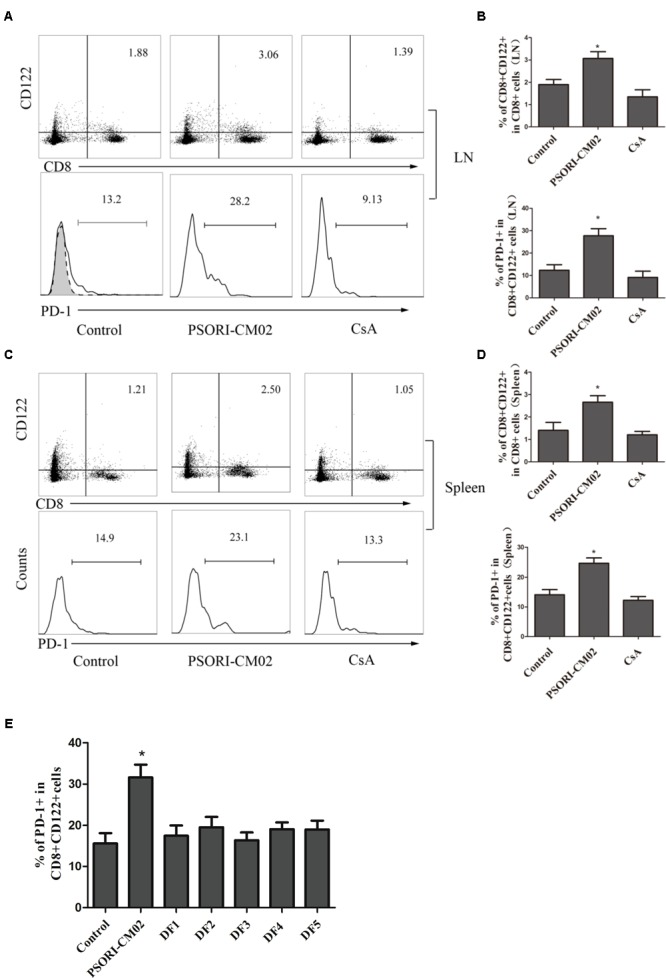
PSORI-CM02 induces CD8+CD122+PD-1+ Tregs. LN and spleen cells were isolated from B6 mice that were transplanted with Balb/C skin and treated with high doses of PSORI-CM02 for 2 weeks. Cells were stained for CD8, CD122, and PD-1 markers, and histograms were gated on CD8+CD122+ subset. CD8+CD122+ or CD8+CD122+PD-1+ Tregs from LNs **(A,B)** or spleens **(C,D)** were analyzed. One representative of three separate experiments is shown. CD8+CD122+ or CD8+CD122+PD-1+ Treg percentages in LN/spleen cells were presented as mean ± SD from 4 to 6 mice per group **(B,D)**. Also analyzed were percentages of PD-1+ within CD8+CD122+ cells from LNs and spleens of recipient mice that were treated with full PSORI-CM02 or each decomposed formula (DF 1-5) missing one herb per formula **(E)**. One of three separate experiments is shown with 4–5 mice per group (^∗^*P* < 0.05).

Tregs are essential for suppression of allograft rejection (Qin et al., 1993; Zhai and Kupiec-Weglinski, 1999; Fu et al., 2004). We asked whether PSORI-CM02 formula would inhibit allograft rejection by inducing CD4+FoxP3+ and/or CD8+CD122+PD-1+ Tregs (Dai et al., 2010; Li et al., 2014; Liu et al., 2015). Draining LN and spleen cells were isolated and both subsets of Tregs were enumerated by FACS analyses 2 weeks following skin transplantation and treatments with PSORI-CM02. As shown in **Figure 3**, CsA significantly reduced the percentage of CD4+FoxP3+ Tregs in both draining LNs and spleens (mean ± SD = 1.63 ± 0.20 vs. 2.35 ± 0.15, *P* < 0.05, % in spleens; and 2.57 ± 0.28 vs. 4.30 ± 0.45, *P* < 0.05, % in LNs) whereas PSORI-CM02 did not significantly alter CD4+FoxP3+ Treg frequencies when compared to the control group (mean ± SD = 2.37 ± 0.34 vs. 2.35 ± 0.15, *P* > 0.05, % in spleens; and 4.06 ± 0.56 vs. 4.30 ± 0.45, *P* > 0.05, % in LNs) (**Figure 3**). We then examined if PSORI-CM02 would induce CD8+CD122+PD-1+ Tregs, another essential Treg subset (Rifa’i et al., 2004; Endharti et al., 2005; Dai et al., 2010). As shown in **Figures 4A,B**, PSORI-CM02 significantly increased the percentage of CD8+CD122+ cells within CD8+ subset in draining LNs of recipient mice (mean ± SD = 2.80 ± 0.35% vs. 1.70 ± 0.20%, *P* < 0.05). PD-1+ Treg frequency within CD8+CD122+ population (CD8+CD122+PD-1+ Tregs) was also increased in draining LNs of recipient mice treated with PSORI-CM02 (**Figures 4A,B**) (mean ± SD = 18.00 ± 0.90% vs. 10.30 ± 0.98%, *P* < 0.05). Similarly, PSORI-CM02 augmented the frequencies of both CD8+CD122+ (mean ± SD = 2.66 ± 0.29% vs. 1.40 ± 0.35%, *P* < 0.05) and CD8+CD122+PD-1+ Tregs (mean ± SD = 24.63 ± 1.79% vs. 14.10 ± 1.74%, *P* < 0.05) in the spleens of recipient mice (**Figures 4C,D**). Interestingly, the decomposed formulas with only four herbs did not increase CD8+CD122+PD-1+ Tregs (**Figure 4E**). Similar findings also were seen 4 weeks after skin transplantation (data not shown). Moreover, PSORI-CM02 formula, but not the decomposed formulas, increased PD-1+ frequency within CD8+CD122+ cells *in vitro* (**Table 2**). Therefore, PSORI-CM02 induced CD8+CD122+PD-1+, but not CD4+FoxP3+, Tregs.

**Table 2:** PSORI-CM02, but not the decomposed formula of four herbs, induces CD8+CD122+PD-1+ Tregs *in vitro*.

Formula	*N*	Mean ± SD (% PD-1+ cells)	*P*-value
Control	4	10.1 ± 0.9	NA
Full PSORI-CM02: CR, RPR, RSG, MF, SR	4	17.8 ± 2.1	<0.05^∗^
Decomposed formula 1: CR, RPR, RSG, MF	5	10.7 ± 1.1	>0.05
Decomposed formula 2: CR, RPR, RSG, SR	5	11.1 ± 1.3	>0.05
Decomposed formula 3: CR, RPR, MF, SR	4	10.5 ± 0.9	>0.05
Decomposed formula 4: CR, RSG, MF, SR	4	9.6 ± 0.8	>0.05
Decomposed formula 5: RPR, RSG, MF, SR	5	11.4 ± 1.5	>0.05

*FACS-sorted CD8+CD122+ cells were cultured and stimulated with anti-CD3/CD28 Abs in the absence or presence of full PSORI-CM02 or each decomposed formula for 96 h. Cells were stained for CD8, CD122, and PD-1 surface markers, and percentages of PD-1+ Tregs within CD8+CD122+ populations were analyzed via FACS. Data were presented as mean ± SD. One of three separate experiments is shown. ^∗^*P* < 0.05 compared to control.*

### PSORI-CM02 Enhances the Capacity of CD8+CD122+PD1+ Tregs to Suppress Allograft Rejection and T Cell Proliferation in Rag1-/- Mice

**FIGURE 5:**
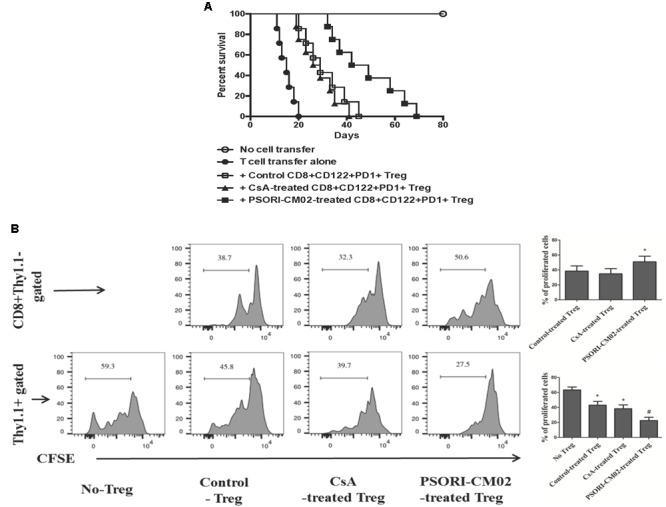
PSORI-CM02-induced CD8+CD122+PD-1+ Tregs prolong skin allograft survival and inhibit T cell proliferation in Rag1-/- recipients. CD8+CD122+PD-1+ Tregs were isolated from B6 recipients that were transplanted with Balb/C skin and treated with CsA or PSORI-CM02. These Tregs (1 × 10^6^), together with purified CD122-CD3+ T cells (4 × 10^6^) from naïve B6 mice at ratios of 1:4, were adoptively transferred to Rag1-/- mice (B6) that were then transplanted with Balb/C skin. Skin allograft rejection (*n* = 7–8 transplants per group) was analyzed **(A)**. To further determine the capacity of the CD8+ Tregs to inhibit T cell proliferation in a transplant setting, CD3+ T cells derived from naïve Thy1.1+ mice (B6) and CD8+CD122+PD-1+ Tregs isolated from CsA- or PSORI-CM02-treated recipient B6 mice were labeled with CESF before they were transferred to Rag1-/- mice that then received skin allografts from BALB/c donors. Treg/T-cell proliferation finally was analyzed via FACS **(B)**. Histograms were gated on the population of CD8+Thy1.1- Tregs or Thy1.1+ T cells. Data are presented as mean ± SD from 4 to 5 mice per group. Shown is a representative of three separate experiments with similar results. (^∗^ represents comparisons with either control Treg [upper panel] or no Treg at all [lower panel], both *P* < 0.05; and ^#^ indicates comparisons with either control Treg or CsA-treated Treg).

Given that PSORI-CM02 augmented CD8+CD122+PD-1+ Treg frequency in recipients, we asked whether PSORI-CM02 also enhanced their suppressive function. CD8+CD122+PD-1+ Tregs were isolated from B6 mice that were transplanted with Balb/C skin and treated with CsA or PSORI-CM02. These Tregs, together with CD3+ T cells isolated from naïve B6 mice, were adoptively transferred to Rag1-/- (B6) recipients that were then transplanted with a Balb/C skin graft. As shown in **Figure 5A**, transfer of CD3+ T cells resulted in allograft rejection in Rag1-/- recipients while transfer of both CD8+CD122+PD-1+ Tregs and CD3+ T cells prolonged allograft survival compared to transfer of CD3+ T cells alone (MST = 29 vs. 15 days, *P* < 0.05). Importantly, PSORI-CM02-induced Tregs further extended allograft survival compared with control Tregs (MST = 46 vs. 29 days, *P* < 0.05) or CsA-treated Tregs (MST = 46 vs. 27 days, *P* < 0.05) (**Figure 5A**), suggesting that PSORI-CM02 enhances suppressor function of CD8+CD122+PD-1+ Tregs. To further determine the capacity of the CD8+ Tregs to proliferate and inhibit T cell proliferation in this model, CD3+ T cells derived from naïve Thy1.1+ mice (B6) and CD8+CD122+PD-1+ Tregs isolated from PSORI-CM02-treated recipient mice were labeled with CESF before they were transferred to Rag1-/- mice that then received skin allografts also from BALB/c mice. Treg/T-cell proliferation was analyzed via FACS. As shown in **Figure 5B**, the Thy1.1-CD8+ Tregs derived from PSORI-CM02-treated recipients proliferated more vigorously than those from control or CsA-treated recipients (mean ± SD = 50.9 ± 7.55% vs. 34.87 ± 6.89% or 50.9 ± 7.55% vs. 38.40 ± 6.96%, *P* < 0.05) while they also suppressed Thy1.1+ T cell proliferation more potently than those from control or CsA-treated recipients (mean ± SD = 22.50 ± 4.37% vs. 43.03 ± 5.05% or 22.50 ± 4.37% vs. 38.20 ± 5.21%, *P* < 0.05).

### PSORI-CM02 Suppresses T Cell Proliferation *in Vitro* While Increasing IL-10 Production

**FIGURE 6:**
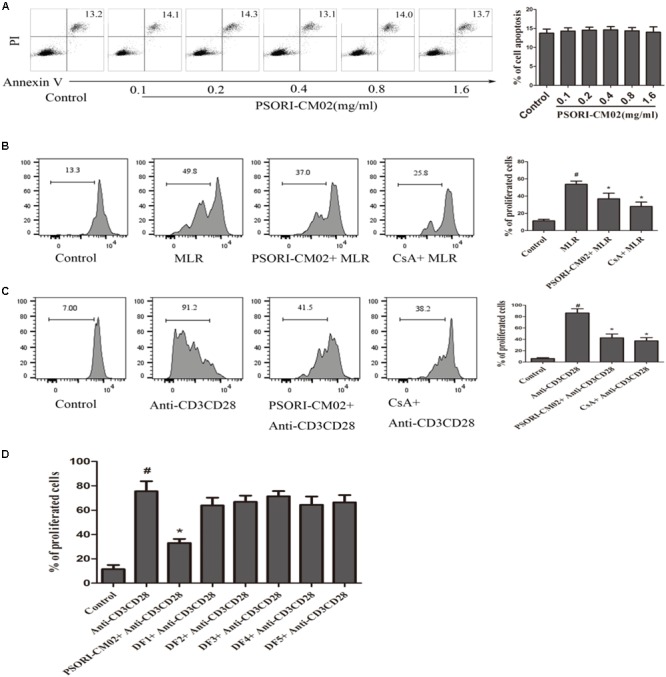
PSORI-CM02 suppresses T cell proliferation *in vitro*. Apoptotic rates of T cells that were treated with anti-CD3/anti-CD28 Abs and PSORI-CM02 for 96 h were determined via FACS analyses **(A)**. Shown also was a representative of CFSE-labeled T cell proliferation following culture with PSORI-CM02 or CsA in one-way MLRs for 96 h **(B)**. One-way MLRs were set up using FACS-sorted CD3+ T cells from B6 mice as responders and irradiated donor Balb/C spleen cells as stimulators. Also shown was the proliferation of CFSE-labeled T cells treated with anti-CD3/anti-CD28 Abs plus PSORI-CM02, CsA **(C)** or decomposed formula 1-5 (DF 1-5) vs. full PSORI-CM02 for 96 h **(D)**. Data are presented as mean ± SD from 4 to 5 wells or samples per group. One representative of three independent experiments with similar results is shown. (^#^ represents comparisons with control, *P* < 0.05 and ^∗^ represents comparisons with MLR or anti-CD3/CD28 alone, *P* < 0.05).

**FIGURE 7:**
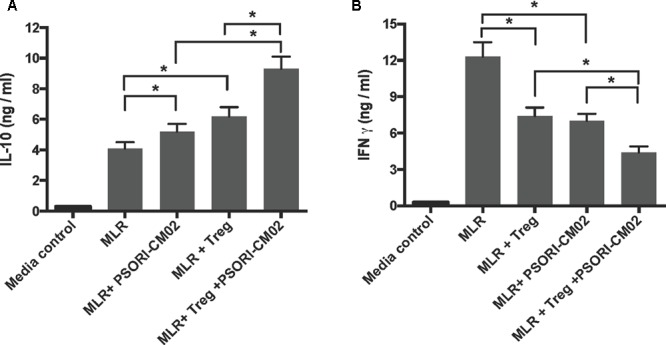
PSORI-CM02 and CD8+CD122+PD-1+ Tregs synergize to increase IL-10 but reduce IFNγ production in the supernatant of MLRs. One-way MLRs were set up using FACS-sorted CD3+ T cells from B6 mice as responders and irradiated donor Balb/C spleen cells as stimulators, as described in the Materials and Methods section. CD8+CD122+PD1+ Tregs, at the ratios of 1:4 (Tregs: Responders), was added to the cell culture in the presence or absence of PSORI-CM02 for 96 h. IL10 **(A)** and IFNγ **(B)** in the supernatant were measured via ELISA. Data are presented as mean ± SD from 4 to 5 wells or samples per group. One representative of three independent experiments with similar results is shown (^∗^*P* < 0.05).

Since PSORI-CM02 inhibited skin allograft rejection, we then asked whether it suppressed T cell proliferation *in vitro* and altered cytokine production. FACS-sorted and B6-derived CD3+ T cells were labeled with CFSE and stimulated with irradiated Balb/C splenocytes (MLR) or anti-CD3+anti-CD28 Abs (anti-CD3/CD28) in the presence of PSORI-CM02 for 96 h. We first determined its potential cytotoxicity. As shown in **Figure 6A**, PSORI-CM02, at concentrations of 0.1–1.6 mg/ml, had no cytotoxic effects 96 h after stimulation with anti-CD3/anti-CD28 Abs as apoptotic rates of the cells were not altered at all. Hence, a concentration of 1 mg/ml for PSORI-CM02 was chosen as a treatment concentration for *in vitro* T cell proliferation assays. As shown in **Figure 6B**, either PSORI-CM02 or CsA suppressed T cell proliferation in a setting of MLR (mean ± SD = 36.97 ± 6.45% vs. 53.77 ± 3.74% or 28.03 ± 5.09% vs. 53.77 ± 3.74%, *P* < 0.05). PSORI-CM02 or CsA also exhibited strong suppression of T cell proliferation when cells were stimulated by anti-CD3 plus anti-CD28 Abs (mean ± SD = 42.62 ± 6.68% vs. 86.43 ± 7.32% or 37.36 ± 5.67% vs. 86.43 ± 7.32%, *P* < 0.05) (**Figure 6C**). Importantly, full PSORI-CM02 formula inhibited T cell proliferation whereas all of the decomposed formula (DF) with a differential combination of four herbs failed to significantly suppress T cell proliferation (**Figure 6D**). On the other hand, PSORI-CM02 moderately increased IL-10 level in the supernatant of the MLRs with a statistical significance (mean ± SD = 5.3 ± 0.5 vs. 4.1 ± 0.4, *P* < 0.05) while CD8+CD122+PD-1+ Tregs significantly augmented IL-10 level (mean ± SD = 6.2 ± 0.6 vs. 4.1 ± 0.4, *P* < 0.05) (**Figure 7A**). PSORI-CM02 further promoted IL-10 production in the presence of the Tregs (mean ± SD = 9.3 ± 0.8 vs. 6.2 ± 0.6, *P* < 0.05). However, either the Tregs or PSORI-CM02 reduced IFNγ in the supernatant (Tregs: 7.4 ± 0.7 vs. 12.3 ± 1.1 and PSORI-CM02: 7.0 ± 0.6 vs. 12.3 ± 1.2, both *P* < 0.05) (**Figure 7B**).

### PSORI-CM02 Inhibits the Signaling Pathways of mTOR and NFκB in Activated T Cells

**FIGURE 8:**
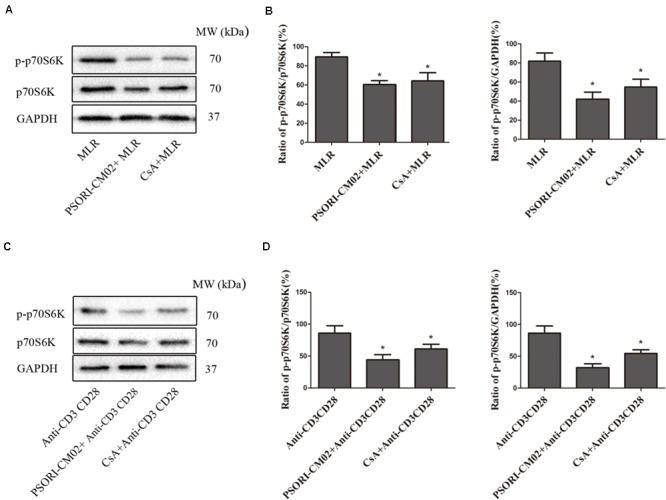
PSORI-CM02 suppresses the phosphorylation of P70S6K in activated T cells. One-way MLRs were set up using T cells isolated from B6 mice as responders and irradiated donor Balb/C spleen cells as stimulators. Expression of p70S6K and phosphorylated p70S6K (p-p70S6K) was detected using Western blotting 72 h after cell culture **(A,B)**. Expression of p70S6K and p-p70S6K by T cells was also detected using Western blotting 72 h after stimulation with anti-CD3 plus anti-CD28 Abs **(C,D)**. One representative of three sets of blotting images is shown. The densitometry analyses of the immunoblotting results are also shown **(B,D)**. Data are presented as mean ± SD. (^∗^ represents comparisons with MLR or anti-CD3/CD28 control group, *P* < 0.05).

**FIGURE 9:**
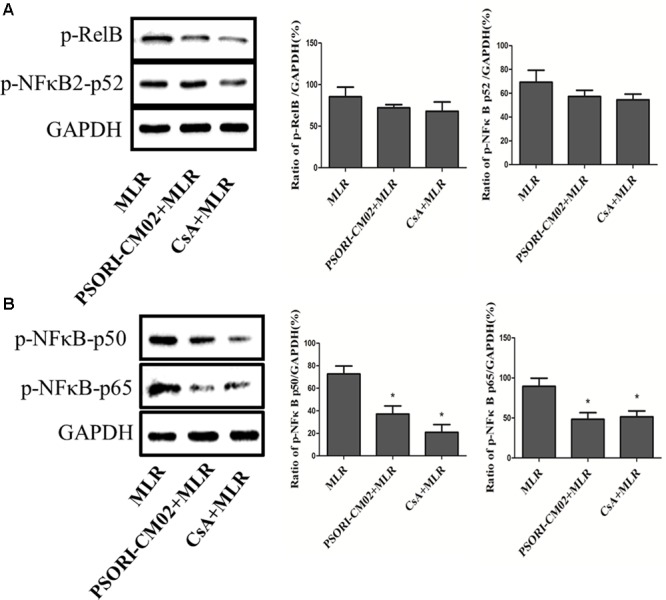
PSORI-CM02 inhibits the phosphorylation of P50/65 in activated T cells. One-way MLRs were set up using T cells isolated from B6 mice as responders and irradiated donor Balb/C spleen cells as stimulators. Expression of phosphorylated Rel B/p52 **(A)** and p50/p65 **(B)** was detected using Western blotting 72 h after the cells were treated with PSORI-CMO2 or CsA. One representative of three separate sets of blotting images is shown. Data are presented as mean ± SD of percentages relative to GAPDH (^∗^*P* < 0.05 compared to MLR alone).

Given that PSORI-CM02 inhibited T cell proliferation *in vitro*, we asked whether it alters the phosphorylation of mTOR signaling. Purified T cells were activated *in vitro* in a MLR setting or through stimulation with anti-CD3 and anti-CD28 Abs. Expression of total p70S6K or phosphorylated p70S6K (p-p70S6K) was detected using Western blotting 72 h after culture. As shown in **Figures 8A,B**, PSORI-CM02 dramatically reduced the phosphorylation of p70S6K in an MLR setting (mean ± SD = 41.93 ± 7.55% vs. 81.86 ± 8.56%, *P* < 0.05). It also suppressed p70S6K phosphorylation when T cells were stimulated with anti-CD3 plus anti-CD28 Abs (**Figures 8C,D**) (mean ± SD = 32.00 ± 6.18% vs. 86.42 ± 11.20%, *P* < 0.05), suggesting that PSORI-CM02 inhibits mTOR signaling during T cell activation. On the other hand, we also determined the effects of PSORI-CM02 on T-cell NFκB signaling in an MLR setting. As shown in **Figure 9**, we found that it reduced the phosphorylation of P50 (mean ± SD = 37.16 ± 7.16% vs. 72.69 ± 6.98%, *P* < 0.05) and P65 (mean ± SD = 48.26 ± 8.35% vs. 89.53 ± 10.17%, *P* < 0.05), but not P52 (mean ± SD = 57.26 ± 5.29% vs. 69.40 ± 9.97%, *P* > 0.05). The latter is an NFκB2 signaling pathway.

## Discussion

Traditional Chinese medicine has been widely used to treat diseases for over 2,000 years. However, understanding of its mechanisms responsible for its therapeutic effects has been hampered due to the complexity and redundancy of TCM, which has limited its worldwide application in clinic. It is generally difficult to provide sound scientific evidence regarding why a TCM formula requires several or even dozens of herbs for it to take effect. Thus, it is compelling to explore its mechanisms of action in order to further expand its clinical application.

PSORI-CM01 formula, also known as Yin Xie Ling with seven herbs, has become a classically formulated Chinese medicine for treatments of psoriasis without causing any major side effect for a decade (Lu et al., 2012; Parker et al., 2014). Recent studies have demonstrated that PSORI-CM01 has an anti-inflammatory effect and can also inhibit the production of proinflammatory cytokines and chemokines (Parker et al., 2014; Wei et al., 2016; Han et al., 2017). In order to reduce its complexity and redundancy, we decided to “sharpen” this old formula. Based on the theory of TCM, we omitted two non-essential herbs, Liquorice and Lithospermum, but retained the rest of five herbs in PSORI-CM01 formula: Rhizoma curcumae, Radix paeoniae rubra, Sarcandra glabra, Rhizoma smilacis glabrae, and Fructus mume, and renamed the new formula as PSORI-CM02. PSORI-CM02 formula itself did not contain CsA or rapamycin, a typical immunosuppressant. We found that PSORI-CM02 suppressed T cell proliferation and prolonged skin allograft survival. Interestingly, this new formula neither extended skin allograft survival nor suppressed T cell proliferation when any single herbal component was omitted (**Table 1**), suggesting that PSORI-CM02 exerts its effects through sound cooperation between these five herbal components, not just via the action of one to four molecular ingredients in the formula. It remains unknown which molecules in the formula work together to exert its suppression of allograft rejection. This is a pitfall for this current study. However, it is nearly impossible to find out exactly how many molecules are needed in order for the formula to become effective since it may contain several hundred of chemicals. Interactions between dozens of molecules within the formula could be involved. In our view, the actual inhibition of allograft rejection by the formula without occurrence of any major side effect is more valuable than merely attempting to identify exactly how many molecules in the formula are responsible for its net effects. To our knowledge, we provided the first scientific evidence, based on modern biomedical studies instead of the theory of TCM, that a typical TCM formula requires at least several herbs to work together to take effect. Thus, results-oriented TCM developed over several centuries may provide a fast track to modern drug discoveries.

CD4+CD25+FoxP3+ Tregs play a critical role in the maintenance of immune tolerance by suppressing aggressive T cell responses. Previous studies have shown that induction of endogenous Tregs or adoptive transfer of exogenous Tregs prevents autoimmune diseases and suppresses allograft rejection in animal models (Qin et al., 1993; Asano et al., 1996; Suri-Payer et al., 1998; Itoh et al., 1999; Zhai and Kupiec-Weglinski, 1999; Shevach, 2000; Hoffmann et al., 2002; Fu et al., 2004). Hence, we determined if PSORI-CM02 inhibited allograft rejection by inducing CD4+FoxP3+ Tregs. Surprisingly, we found that PSORI-CM02 did not alter CD4+FoxP3+ Treg percentages in lymph nodes and spleens while CsA reduced their frequencies. Zhao et al. (2012) demonstrated that curcumin, an ingredient also obtained from Sarcandrae herba, inhibited the suppressive capacity of CD4+CD25+ Tregs by reducing nuclear translocation of NFκB (Zhao et al., 2012). It is likely that the net effects of PSORI-CM02 on the Tregs may be attributed to the interactions between many molecules rather than curcumin alone. In fact, some studies have shown that curcumin suppresses autoimmunity and GVHD by inducing or expanding CD4+FoxP3+ Tregs (Park et al., 2013; Zhao et al., 2016; Ohno et al., 2017). On the other hand, CsA has been widely used to treat autoimmune diseases and allograft rejection. However, previous studies have demonstrated that CsA hinders the generation and function of CD4+CD25+ Tregs (Baan et al., 2005; Shibutani et al., 2005; Wang et al., 2006), possibly via impeding IL-2 expression (Zeiser et al., 2006; Kang et al., 2007; Noris et al., 2007). Furthermore, CsA may also cause additional side effects, including nephrotoxicity and infections. Therefore, PSORI-CM02 appears to be promising for tolerance induction compared to CsA since the former does not repress CD4+FoxP3+ Tregs.

NFκB pathway appears to play dual roles in Treg development and induction. On one hand, it was required for Treg development, especially in thymi (Long et al., 2009). Previous studies also demonstrated that curcumin inhibited the suppressive activity of CD4+CD25+ Tregs by reducing nuclear translocation of p65 and c-Rel (Zhao et al., 2012). On the other hand, recent studies revealed that NFκB signaling also was needed for the suppression of Treg generation and function (Harusato et al., 2017; Sukumar et al., 2017). In particular, the inhibitor of NFκB (IκB) drives Foxp3 expression (Schuster et al., 2012), suggesting that suppression of NFκB activation is also required for Treg induction. Therefore, the role for NFκB signaling in Treg generation/induction remains controversial. Here, we found that PSORI-CM02 formula reduced P50/65 phosphorylation but did not significantly alter FoxP3+ Treg frequency, indicating that NFκB signaling intensity does not affect the Treg generation in the periphery, at least in the context of transplantation.

Mounting evidence has demonstrated that CD8+CD122+ T cells are another subset of Tregs that inhibit conventional T cell responses (Rifa’i et al., 2004; Endharti et al., 2005, 2011; Chen et al., 2008; Shi et al., 2008; Molloy et al., 2011), antitumor immunity (Wang et al., 2010) and autoimmune responses (Kim et al., 2011; Mangalam et al., 2012), although other subsets of CD8+ Tregs have also been shown to suppress alloimmune responses (Colovai et al., 2000; Koch et al., 2008; Aoyama et al., 2012; Lerret et al., 2012; Zheng et al., 2013; Barbon et al., 2014; Zimmerer et al., 2014). We have also shown that CD8+CD122+ T cells are not only Tregs (Dai et al., 2010; Li et al., 2014; Liu et al., 2015), but also more potent in suppression of allograft rejection than conventional CD4+CD25+ Tregs (Dai Z. et al., 2014). Especially, we have previously found that PD-1-positive component within CD8+CD122+ T cell population is mainly responsible for their regulatory activities (Dai et al., 2010). CD8+CD122+ Tregs likely correspond to their CD4+CD25+ counterparts since CD122 is the β subunit of IL-2 receptor on T cells while CD25 is the α subunit of the same receptor (Sakaguchi et al., 1995). Therefore, both CD4+CD25+ and CD8+CD122+ Tregs are important components of the Treg family. Given that PSORI-CM02 did not alter CD4+CD25+ Tregs in our model, we examined whether PSORI-CM02 induced CD8+CD122+PD-1+ Tregs. We found that PSORI-CM02 indeed augmented CD8+CD122+PD-1+ Treg frequency. Importantly, it also enhanced their capacity to inhibit allograft rejection, indicating that PSORI-CM02 induces CD8+CD122+PD-1+ Tregs quantitatively and qualitatively.

## Author Contributions

CL provided key reagents and experimental design. HL, XJ, and YC performed the experiments and analyzed the data. CL-L and FQ analyzed the data. ZD wrote the manuscript and designed the experiments.

## Conflict of Interest Statement

The authors declare that the research was conducted in the absence of any commercial or financial relationships that could be construed as a potential conflict of interest.

## Abbreviations

CsA: cyclosporine
DC: dendritic cell
HPLC: high performance liquid chromatography
LN: lymph node
MST: median survival time
mTOR: mechanistic target of rapamycin
TCM: traditional Chinese medicine
Treg: regulatory T cell
