# Supplementary material for: Herbal Components of a Novel Formula PSORI-CM02 Interdependently Suppress Allograft Rejection and Induce CD8+CD122+PD-1+ Regulatory T Cells
Source: Front Pharmacol. 2018 Feb 12;9:88. doi: 10.3389/fphar.2018.00088 (PMC5816027; doi:10.3389/fphar.2018.00088)
Supplement: Supplementary file 1 [file Table_1.pdf]

Supplementary Table S1. Biochemical tests of hepatic and renal function

Groups	AST (mmol/L)	ALT (mmol/L)	CRE (mmol/L)
Normal control	21.65±3.45	6.47±0.54	26.15±2.25
Psoriatic control	22.34±2.23	6.63±0.48	25.94±4.17
PSORI-CM02-L	24.31±1.12	5.48±0.77	25.61±4.56
PSORI-CM02-H	23.34±2.48	7.19±0.66	27.16±5.13
Tripterygium wilfordii	59.11±6.69*	17.45±1.33*	64.06±7.64*

(AST: Aspartate transaminase; ALT: Alanine transaminase; and CRE: Creatinine)

Recipient mice were treated with PSORI-CM02-L (2 g/kg/day) or PSORI-CM02-H (6 g/kg/day). Four weeks later, serum AST, ALT and CRE were measured by Department of Laboratory Medicine, Guangdong Provincial Hospital of Chinese Medicine (Guangzhou, China). Higher doses of Tripterygium wilfordii (15 g/kg/day) served as a positive control. Data are presented as Mean SD. Six to eight mice per group were derived from two separate experiments (* P<0.05 compared to control groups).
