# Supplementary material for: Herbal Components of a Novel Formula PSORI-CM02 Interdependently Suppress Allograft Rejection and Induce CD8+CD122+PD-1+ Regulatory T Cells
Source: Front Pharmacol. 2018 Feb 12;9:88. doi: 10.3389/fphar.2018.00088 (PMC5816027; doi:10.3389/fphar.2018.00088)
Supplement: Supplementary file 2 [file Image_1.pdf]

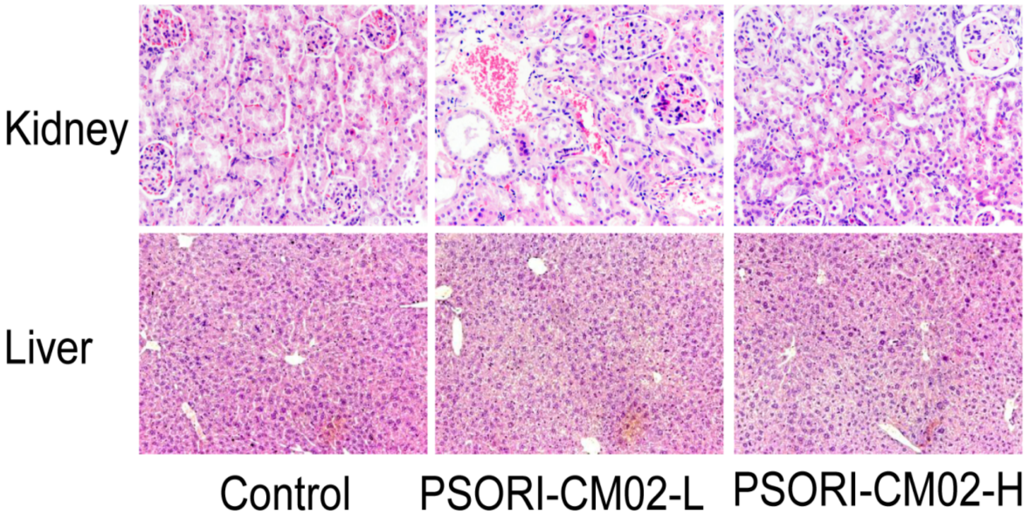

Supplementary Figure S1: Administration of PSORI-CM02 does not result in any toxic injury to a murine kidney or liver

HE staining was performed on tissue sections of kidneys and livers derived from transplanted recipient mice that were treated with PSORI-CM02-L (2 g/kg/day) or PSORI-CM02-H (6 g/kg/day) for four weeks. One representing set from three separate experiments is shown.
