# Supplementary material for: Herbal Components of a Novel Formula PSORI-CM02 Interdependently Suppress Allograft Rejection and Induce CD8+CD122+PD-1+ Regulatory T Cells
Source: Front Pharmacol. 2018 Feb 12;9:88. doi: 10.3389/fphar.2018.00088 (PMC5816027; doi:10.3389/fphar.2018.00088)
Supplement: Supplementary file 3 [file Image_2.pdf]

A

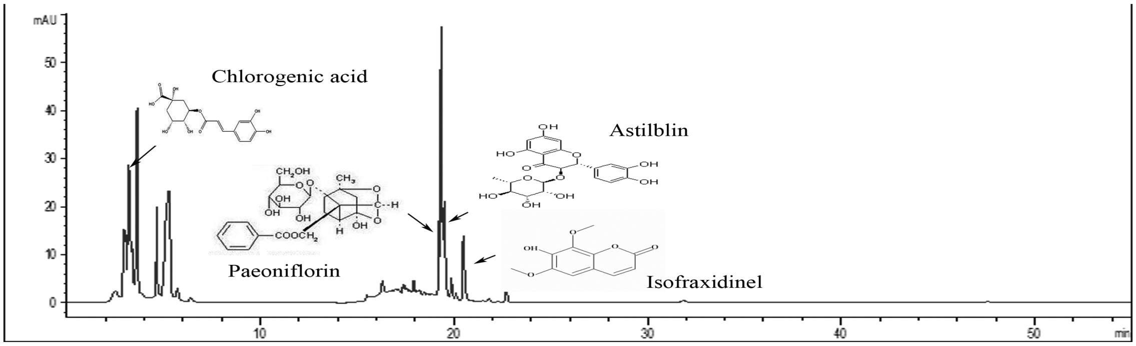

B

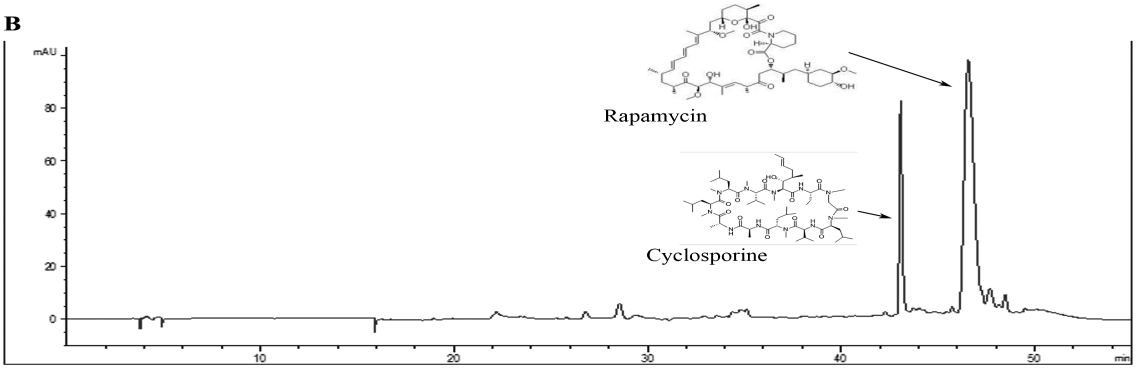

Supplementary Figure S2: PSORI-CM02 does not contain conventional an immunosuppressant cyclosporine or rapamycin

PSORI-CM02 formula and all standard samples (**A**), including Chlorogenic acid, Astilbin, Isofraxidine and Paeoniflorin, as well as immunosuppressive agents CsA and rapamycin (**B**) were subject to HPLC analysis using an Agilent 1200 HPLC system consisting of C18 pump. One of two independent experiments is shown. The results suggest that PSORI-CM02 formula does not contain a conventional immunosuppressant CsA or rapamycin.
